# Important denominator between autoimmune comorbidities: a review of class II HLA, autoimmune disease, and the gut

**DOI:** 10.3389/fimmu.2023.1270488

**Published:** 2023-09-26

**Authors:** Meghan A. Berryman, Jorma Ilonen, Eric W. Triplett, Johnny Ludvigsson

**Affiliations:** ^1^ Triplett Laboratory, Institute of Food and Agricultural Sciences, Department of Microbiology and Cell Science, University of Florida, Gainesville, FL, United States; ^2^ Immunogenetics Laboratory, Institute of Biomedicine, University of Turku, Turku, Finland; ^3^ Crown Princess Victoria’s Children’s Hospital and Division of Pediatrics, Department of Biomedical and Clinical Sciences, Linköping University, Linköping, Sweden

**Keywords:** ABIS, type 1 diabetes, celiac disease, rheumatoid arthritis, autoimmune thyroid disease, HLA-DR, HLA-DQ

## Abstract

Human leukocyte antigen (HLA) genes are associated with more diseases than any other region of the genome. Highly polymorphic HLA genes produce variable haplotypes that are specifically correlated with pathogenically different autoimmunities. Despite differing etiologies, however, many autoimmune disorders share the same risk-associated HLA haplotypes often resulting in comorbidity. This shared risk remains an unanswered question in the field. Yet, several groups have revealed links between gut microbial community composition and autoimmune diseases. Autoimmunity is frequently associated with dysbiosis, resulting in loss of barrier function and permeability of tight junctions, which increases HLA class II expression levels and thus further influences the composition of the gut microbiome. However, autoimmune-risk-associated HLA haplotypes are connected to gut dysbiosis long before autoimmunity even begins. This review evaluates current research on the HLA-microbiome-autoimmunity triplex and proposes that pre-autoimmune bacterial dysbiosis in the gut is an important determinant between autoimmune comorbidities with systemic inflammation as a common denominator.

## Graphical Abstract

Graphical representation of central hypothesis.

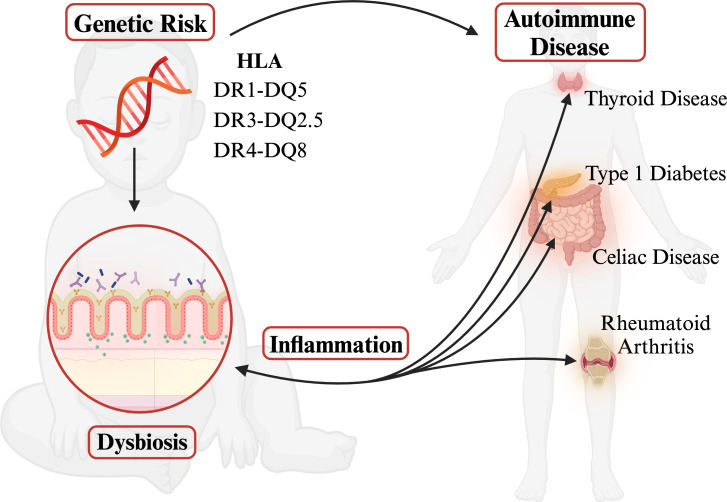

## 1 Introduction

The major histocompatibility complex (MHC) has long been established as the human genetic region associated with the greatest number of autoimmune diseases (1, 2). The MHC is broadly categorized into three classes: class I, which encodes for *HLA-A, HLA-B, HLA-C, HLA-E, HLA-F*, and *HLA-G* genes; class II, the focus of this review, which encodes for *HLA-DR, HLA-DQ*, and *HLA-DP* genes; and class III, which includes components of the complement system, immune regulators, and non-immune associated genes (2–4). Classically, class I HLA are present on all cells, while class II HLA are expressed on the surface of antigen presenting cells (APC) like dendritic cells and macrophages. Class I HLA-peptide combinations bind CD8^+^ T cell αβ T cell receptors (TCRs) for inspection of internally found antigens, like signals of viral infection and cancer. Class II HLA present externally found peptides to CD4^+^ T cell TCRs, such as bacteria and other foreign pathogens. However, cross-class presentation has been observed to bypass MHC restriction (4–6). The presentation of externally found antigens to T cells instigates a cascade leading to destruction of the perceived pathogen. HLA-peptide-TCR interaction specificity is fundamental to an effective cell-mediated adaptive immune response (7). The peptide repertoire available for presentation by class II HLA largely depends on the structure of the binding pocket.

HLA DR and DQ loci are highly polymorphic and exhibit an elevated amount of linkage disequilibrium. The combination of these features contribute to creating distinctive and behaviorally differential HLA haplotypes (8). This review will cover autoimmune-risk-associated class II HLA haplotypes DR4-DQ8, DR3-DQ2, and DR1-DQ5. The most polymorphic regions of the DR and DQ molecules are located within extracellular regions making up the peptide-binding cleft, which cause structure-altering changes at the amino acid level (9–12). These structural variations alter peptide-binding and thus antigen-presenting capabilities (7, 13, 14). The structural differences between haplotype molecules result in unique sensitivities and can be the determining factor for many autoimmune diseases, such as type 1 diabetes (T1D), celiac disease (CD), rheumatoid arthritis (RA), and autoimmune thyroid disease (AITD), including Grave’s disease (GD) and Hashimoto’s disease (HD) (9, 15–18).

Epidemiological data show an increase in the frequency of autoimmune diseases over the past few decades that cannot be explained by genetics alone (19–21). Many autoimmune disorders share the same risk-associated HLA haplotypes often resulting in comorbidity despite having differing etiologies (22–24). The combination of high polymorphism and linkage disequilibrium within the gene dense MHC region leads to difficulty in determining the mechanism for the autoimmune associations observed (1, 2). This gap is where the role of the gut microbiome has become increasingly essential in defining the pathogenesis of these autoimmune diseases (25–30). It has been theorized that the dysbiosis seen in autoimmune diseases is associated with systemic inflammation, resulting in loss of barrier function and permeability of tight junctions, allowing for possible increased exposure of HLA proteins to bacterial antigens (31–33). HLA class II proteins are expressed in the upper villi of small intestinal enterocytes at a steady state in the presence of a healthy gut microbiome and are an integral part of maintaining homeostasis; however, dysbiosis and inflammation cause an increase in HLA class II expression in small intestinal crypts and the colonic epithelium, which can in turn influence the composition of the gut microbiome (32, 34–39). Notably, the increase in HLA class II expression levels is active-disease dependent; for example, celiac patients with exposure to gliadin show HLA upregulation whereas celiac patients in remission have HLA class II levels of controls (40). However, certain HLA haplotypes, specifically the known risk HLA discussed here, are associated with gut dysbiosis before autoimmunity occurs (36, 39, 41, 42). Such evidence suggests that certain HLA may be predisposing an individual to systemic inflammation originating from the gut microbiome by clearing beneficial microbes and creating the potential for dysbiosis early in life. The tripartite HLA-microbiome-autoimmunity link is not trivial. This review summarizes current research on the impact class II HLA haplotypes have on the microbiome and its correlation to autoimmune disease onset. Our hypothesis is that bacterial dysbiosis in the gut leads to systemic inflammation which leads to autoimmunity (Graphical Abstract). The sources and types of inflammation can vary, causing different autoimmune disease outcomes.

## 2 DR-DQ haplotype structure and nomenclature

**Figure 1:**
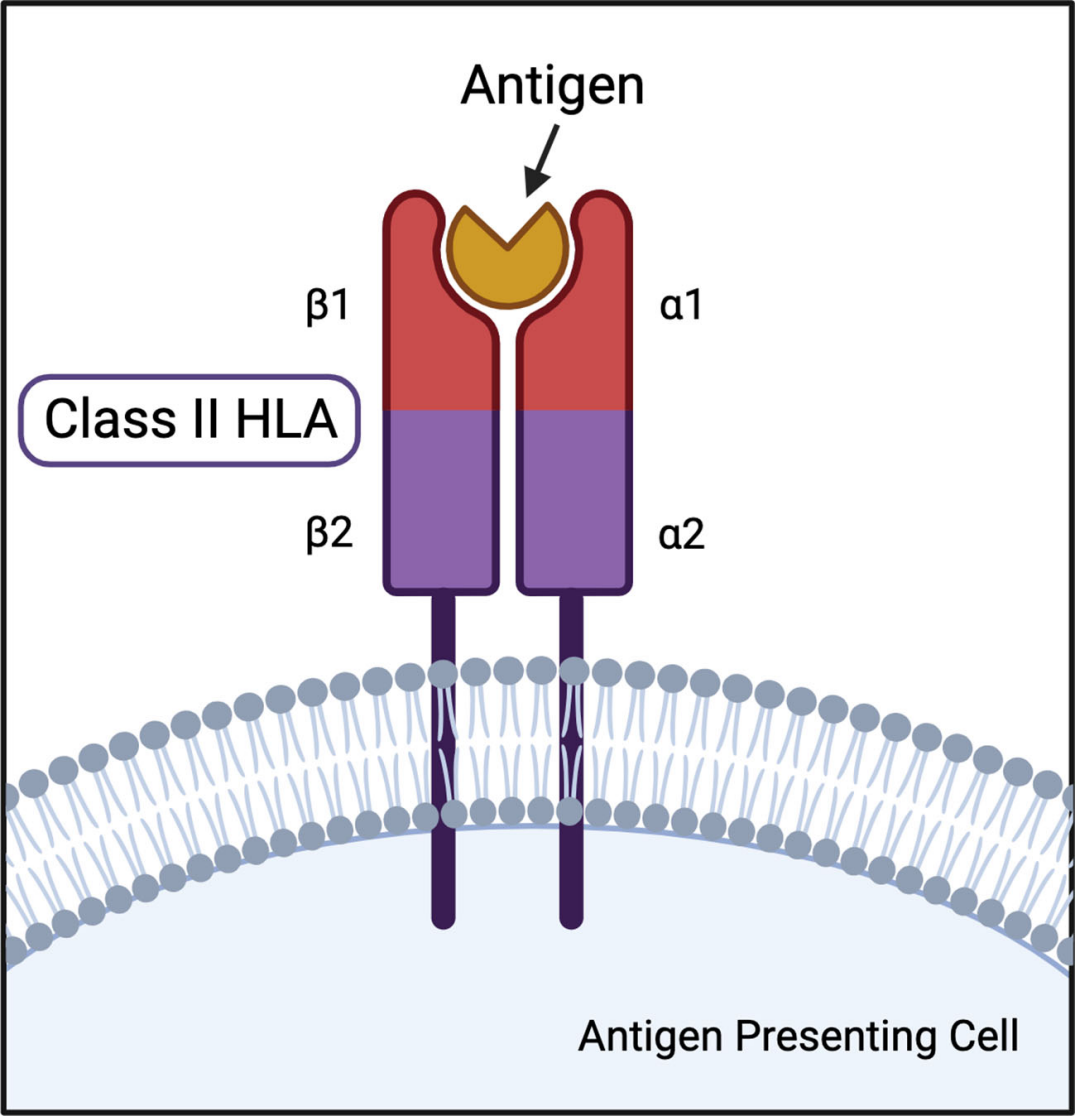
Illustration of class II HLA protein structure with antigen in peptide binding pocket.

Class II HLA DR and DQ loci represent the greatest genetic determinants of multiple autoimmune diseases. HLA-DR is a heterodimer consisting of an α (DRA) and β (DRB) chain, each of which have two extracellular domains, an intramembranous domain, and a cytoplasmic tail (**Figure 1**). DRA has two potential α polypeptide chains for the HLA-DR heterodimer, but the allelic differences do not result in function-altering polymorphisms (43, 44). The HLA-DR β chain can be encoded by *DRB1*, *DRB2* (pseudogene), *DRB3*, *DRB4*, and *DRB5* genes (43). Many DRB1 allelic variations are associated with multiple autoimmune diseases and are the basis for the HLA-DR naming system. For example, HLA-DR4 is the name for the DRB1*04 allele group. HLA-DQ is also a highly variable αβ heterodimer forming a type 1 membrane protein. DQA and DQB can both be encoded by two paralogs: *DQA1, DQA2, DQB1, DQB2*, respectively. Both DQA1 and DQB1 are highly polymorphic resulting in hundreds of possible combinations (43).

HLA DR4-DQ8 is the nomenclature used to represent that an individual has the gene products of HLA *DRA1*-DRB1*04:01/02/04/05/08 and DQA1*03:01-DQB1*03:02/04 (11). DR3-DQ2.5 represents gene products of DRB1*03:01/02/03/04-DQA1*05:01-DQB1*02:01. DR1-DQ5 represents DRB1*01:01/02-DQA1*01:01-DQB1*05:01. DR5-DQ7.5 represents DRB1*05:01-DQA1*05:05-DQB1*03:01. For clarification, the HLA naming system (e.g., DRB1*04:01) is the gene locus name (e.g., *DRB1*), followed by an asterisk, the serologic designation of the allelic group (e.g., 04), a colon, and then the numeric designation of the specific HLA protein (e.g., 01). The naming system can be further expanded to a six-digit identifier that includes another colon followed by a two-digit number that represents a synonymous DNA substitution in the coding region (e.g., DRB1*04:01:01). For this review, the four-digit naming system will be sufficient.

Because *DQA1* and *DQB1* can both have polymorphisms, unique DQ heterodimers can be formed by pairing α and β chains from the same chromosome (*cis*) or opposite chromosomes (*trans*). While the *cis* form of DQ has been studied predominantly, *trans* variants are functional and surface expressed (45). This *trans* molecular formation means that a person heterozygous for DR4-DQ8 and DR3-DQ2.5 can produce a DQ2.3 (DQA1*03:01-DQB1*02:01) molecules from the α chain of DQ8 and the β chain of DQ2.5 (46). For this review, all DQ can be assumed to be *cis* unless specifically reported as *trans.*

## 3 Class II HLA autoimmunity association

### 3.1 Type 1 diabetes

Type 1 diabetes (T1D) is characterized by autoimmune destruction of pancreatic β-cells, resulting in a lifelong exogenous insulin dependency that affects millions of people worldwide (47). While there are over 50 known regions of the genome to show association with genetic risk for T1D, the greatest genetic determinants are MHC class II *DRB1*, *DQA1*, and *DQB1* (48). The haplotypes most strongly associated with T1D susceptibility in those with European ancestry are DRB1*04:01-DQA1*03:01-DQB*03:02, DRB1*04:05-DQA1*03:01-DQB1*03:02, and DRB1*04:02-DQA1*03:01-DQB1*03:02, and then DRB1*04:04-DQA1*03:01-DQB1*03:02 conferring weaker disease susceptibility (9, 49). These well-known associations have predominantly been studied in European-centric populations and they may not be translatable to other ethnic backgrounds. For example in the Japanese population DR4-DQ8 is not prevalent; DRB1*04:05-DQA1*03:03-DQB1*04:01 (DR4-DQ4) and DRB1*09:01-DQA1*03:02-DQB1*03:03 (DR9-DQ9) are the most susceptible haplotypes for T1D (50). Three haplotypes, DR4-DQ8, DR3-DQ2.5, and DR4-DQ4, which combine the risk haplotypes for Caucasian and Japanese populations are the most highly associated with T1D in the Taiwanese population (51). For the Caucasian population, the development of diabetes-associated autoantibodies and T1D is significantly more common in children with heterozygous HLA DR3-DQ2/DR4-DQ8 compared to homozygous DR4-DQ8/DR4-DQ8 and DR3-DQ2.5/DR3-DQ2.5 (11, 12, 52). In Finland, children with high-risk HLA DR3-DQ2/DR4-DQ8 genotypes have a 45-fold increased risk for T1D compared to those with neutral or protective genotypes (17). The age of T1D diagnosis and type of autoantibody first observed are also strongly associated with HLA genotypes (53, 54). Insulin autoantibodies (IAA) and insulinoma-associated-2 autoantibodies (IA2A) are strongly correlated with DR4-DQ8 (12, 55). Approximately 1 in 20 heterozygous for DR3-DQ2/DR4-DQ8 will be diagnosed with T1D by the age of 15 (11). The proportion of HLA DR3-DQ2/DR4-DQ8 heterozygous T1D subjects decreases with increasing age at diagnosis (12). Seroconversion and development of T1D is associated with specific residues at both the DR and the DQ loci, the motif lysine, alanine, glycine at DRB1 β71, β74, β86 residues, which corresponds with DRB1*04:01, and glutamine, alanine, and aspartic acid at DQ α44, β57, and β135 residues, respectively, which correspond to DQA1*03:01-DQB1*03:02 (56, 57). An alanine at DQ β57 is most strongly associated with T1D (58).

In addition to DR4-DQ8, among the highest risk HLA haplotypes for T1D is DRB1*0301-DQA1*05:01-DQB1*02:01 (DR3-DQ2.5) (9). For the Caucasian population, the development of diabetes-associated autoantibodies is significantly associated with both the homozygous DR3-DQ2.5 and heterozygous DR3-DQ2.5/DR4-DQ8 (11, 12, 52). As previously discussed, the age of T1D diagnosis and type of autoantibody first observed are also strongly associated HLA haplotype; autoantibodies, IAA and IA2A, are associated with DR4-DQ8 and early seroconversion, but glutamic acid decarboxylase autoantibodies (GADA) are found in individuals have later seroconversion and are strongly associated with DR3-DQ2.5 (12, 52–55). Recent evidence suggests that GAD peptides bind to DR3-DQ2.5 molecules and in turn induce CD4+ T cell cytokine expression (59). In addition to the *cis* DR3-DQ2.5/DR4-DQ8 heterodimer, the heterozygote DR3-DQ2.5/DR4-DQ8 in *trans* heterodimer form encoded by DQA1*05:01-DQB1*03:02 is also very high risk for T1D (9). At a young age the risk for T1D is highest with heterozygous HLA-DR3-DQ2.5/DR4-DQ8, approximately 1 in 20 with this HLA haplotype pairing will be diagnosed with T1D by the age of 15 (11). The strongest amino acid association with T1D onset is an alanine at residue DQ β57, which is seen in both DQ2 and DQ8 (58, 60).

### 3.2 Celiac disease

Celiac disease (CD) is autoimmune enteropathy characterized by the immunogenicity of gliadin peptides derived from dietary gluten. The chief HLA determinant of CD development is HLA-DQ (61). While HLA-DQ2.5 discussed in the next section has the highest risk association, DQ8 specifically, DQA1*03:01-DQB1*03:02, represents about 2–10% of the Caucasian CD population (62). The determining factor for HLA-associated CD susceptibility is the preferential binding of negatively charged gliadin-derived glutamate residues to certain binding pockets of DQ molecules, specifically P1 and P9 for DQ8 and P4, P6 and P7 for DQ2.5 and DQ2.2 (61, 63).

Globally, CD seroprevalence is 1.4% and 1.8% in North America. However, a study recently found that the highest prevalence is 3.1% within those from northwest India (64, 65). Within the Caucasian population, DQ2.5 is the predominant HLA predictor of CD susceptibility, approximately 95% of those with CD are positive for DQA1*05:01-DQB1*02:01 (DQ2.5); the remaining CD population has either DQA1*03:01-DQB1*03:02 (DQ8) or DQA1*02:01-DQB1*02:02 (DQ2.2) (66). DQ2.5 also has the strongest risk association with CD within the Indian population (67). DQ2.5 was the greatest CD-associated HLA in Moroccan, Libyan, Greek, and Italian CD populations; however, approximately 73.9%, 80.7%, 81.3%, and 78.2% of the populations, respectively, were DQ2.5 positive (68). The determining factor for HLA-associated CD susceptibility is the preferential binding of negatively charged gliadin-derived glutamate residues to certain binding pockets of DQ molecules, specifically P4, P6 and P7 for DQ2.5 and DQ2.2 (63). While DQ2.5 is the predominant isoform seen in association with CD susceptibility and the majority of the remaining CD population carry DQ8, DQA1*02:01-DQB1*02:02 (DQ2.2), linked to DRB1*07:01 (DR7), represents about 3.4% of the CD population (69). Generally, DQ2.2 is associated with very low CD risk; however, when heterozygous with DQ2.5 or *trans* configuration creates DQ2.5, a high risk association is observed (69, 70). Risk for CD is associated with DQ2.2 predominantly when individuals also carry DR3-DQ2.5 or DR5-DQ7.5 because the DQA1*05:05 of DQ7.5 is nearly identical to the DQA1*05:01 of DQ2.5 which means the DQB1*02:02 of DQ2.2 and DQA1*05:05 of DQ7.5 can make a DQA1*05:05-DQB1*02:02 (DQ2.5) heterodimer (70).

### 3.3 Autoimmune thyroid diseases

Autoimmune thyroid diseases (AITD) include both Graves’ disease (GD), which is an autoimmune hyperthyroidism, and Hashimoto’s disease (HD), which is an autoimmune hypothyroidism. HD is characterized by positive autoantibodies to thyroglobulin and thyroid peroxidase, whereas GD is characterized by autoantibodies against the thyroid stimulating hormone receptor, thyroglobulin, and thyroid peroxidase (71). The chief genetic determinant of GD susceptibility is DRB1*03:04-DQA1*05:01-DQB1*02:01 (DR3-DQ2.5); however, the DQB1*02:01 locus appears to be associated through linkage disequilibrium as opposed to actual influence on susceptibility (18, 72, 73). The strongest amino acid association with GD is an arginine at residue DR β74, which is integral to the binding and presentation of thyroglobulin (18, 72, 74). The positive charge provided by arginine in this positive likely facilitates auto-antigen presentation (75). Though less convincingly, DR3 is also associated risk of HD susceptibility and, in those with HD and T1D, DR3 is responsible for joint susceptibility (18, 75).

### 3.4 Autoimmune arthritis

The association of DRB1*04 and genetic predisposition for rheumatoid arthritis (RA) has been observed since the late 1980s (76, 77). RA is the autoimmune destruction of the synovium in the small joints characterized by the presence of autoantibodies: rheumatoid factor, anti-cyclic citrullinated peptide-2, and anti-carbamylated protein (78, 79). Within the Caucasian population of RA patients, significant associations are seen with DRB1*04:01, DRB1*04:04, DRB1*04:05 and 95% of those with severe arthritis expressed DRB1*04:01 (80, 81). Those homozygous for DR4 have the highest risk association for RA (82). While allotypes of DR4 are high risk for RA, DRB1*04:02 is not associated with the disease (80). The determining factor for HLA-associated RA susceptibility is a positive charge at the DRβ71 amino acid residue—DRB1*04:01 and DRB1*04:04 have a positively charged lysine or arginine, respectively, at this position whereas DRB1*04:02 has a negatively charged glutamic acid (80). It is important to note that while certain DR4 alleles have long been observed in Caucasian RA studies, there is no statistical significance in the prevalence of DR4 in RA patients versus controls within the Iranian population and no association between RA-associated autoantibodies and risk HLA in the Japanese population (79, 83).

For RA patients who lack DR4, DRB1*01:01/02 (DR1) is notably associated with RA susceptibility (77). Like DRB1*04:01/04, DRB1*0101 carries a positively charged arginine at the DRβ71 amino acid residue, which is a determining factor for HLA-associated RA susceptibility (80). A small study shows that the majority multi-drug resistant RA patients have DRB1*01:01/02 (84). HLA-DRB1*04:01, DRB1*04:05, and DRB1*01:01 share a common motif at residues β11, β13, β71, and β74, specifically an alanine at position 74 and a positively charged lysine or arginine at position 71, influencing the DRB1 P4 binding pocket (85). Also, homozygote DR1-DQ5 is also strongly associated with juvenile idiopathic arthritis (JIA) with an odds ratio of 3.6, which increases to 6.4 when individual was breastfed for fewer than 8 months (86). JIA was also associated with DR5-DQ7 in individuals who breastfed under 8 months (86).

## 4 Autoimmune comorbidity

Despite differing etiologies, as discussed, many autoimmune disorders share the same risk-associated HLA haplotypes often resulting in comorbidity. Individuals with T1D are 4.9 times more likely to have RA as adults than the general population (87). A 2011 study suggests that 12.3% of the T1D population assessed have AITD and 24.6% have CD (88). However, that number was an overestimation. A 2023 study found that while 18.6% of the T1D population tests positive for CD, 12.6% were serologically false positive and only 6% are actually confirmed CD patients (89), which is in agreement with prevalence found in many other studies (90). Globally, biopsy-confirmed CD prevalence is 0.7%; however, biopsy-confirmed CD prevalence is 1.6% in the general AITD population and 2.6% in the hyperthyroid community (64, 91). For those with CD, 26% of the population have AITD compared to 2–5% of the general population; individuals with CD are 2.4 times more likely to develop an AITD and 5.9 times more likely if they are female (92, 93). The odds of having RA is also higher in CD, occurring nearly 2 times as often compared to the general population (94).

## 5 Evidence for autoimmunity-associated dysbiosis

### 5.1 Type 1 diabetes

While T1D is caused by autoreactive T cells, a link between T1D and notable microbial patterns and intestinal inflammation is evident (36, 95, 96). The microbiome of T1D children lack diversity and have higher levels of butyrate-producing and mucin-degrading bacteria than healthy children (38, 97). Microbiome differences of those with future T1D diagnosis can be seen as early as one year of age (42). Research from the Finnish Type 1 Diabetes Prediction and Prevention Study (DIPP), a prospective, general-population cohort, shows high abundance of *Bacteroides dorei* and *Bacteroides vulgatus* between 12 and 15 months before seroconversion (37). Active T1D cases are associated with higher relative abundance of *Ruminococcus* and *Prevotella copri* and lower relative abundance of *Bifidobacterium, Lactobacillus, Roseburia*, and *Faecalibacterium* (**Table 1**) (98–100). The microbial composition observed in T1D patients likely leads to intestinal permeability, causing intestinal inflammation (101). Intestinal permeability results in increased exposure of intestinal immune cells to bacteria antigens. Intestinal biopsies from T1D children showed an increase in class II HLA molecule expression, and high levels of CD25+ cells (102). Increased exposure to commensal bacteria and excessive immune response over time could result in aberrant self-tolerance mechanisms. T1D patients exhibit immune dysregulation with higher percentages of Th1, Th17, and TNFa+ T cells (103).

**Table 1:** Autoimmune disease association with HLA genetics, bacteria, and each other.

Disease	Genetic Risk (HLA)	Comorbidity	Positive Association with Disease	Negative Association with Disease
Autoimmune Arthritis	DR4-DQ8 DR1-DQ5 DR5-DQ7 (JIA specific)	T1D, CD	*Collinsella, Eggerthella, Faecalibacterium, Prevotella copri*	*Bifidobacterium, Bacteroides*
Autoimmune Thyroid Disease	DR3-DQ2.5	CD, T1D	*Lactobacillus, Bacteroides fragilis*	*Lactobacillus, Bacteroides fragilis*
Celiac Disease	DR3-DQ2.5 DR4-DQ8 DR7-DQ2.2 (with DR3-DQ2.5 or DR5-DQ7)	AITD, RA, T1D	*Dialister invisus*, *Parabacteroides* sp., *Porphyromonas* sp., *Ruminococus bicirculans*, Lachnospiraceae, Veillonellaceae, Pasteurellaceae	*Bifidobacterium, Lactobacillus*
Type 1 Diabetes	DR4-DQ8 DR3-DQ2.5	RA, AITD, CD	*Ruminococcus, Prevotella copri*, *Bacteroides dorei*, *Bacteroides vulgatus*	*Bifidobacterium, Lactobacillus, Roseburia, Faecalibacterium*

AITD, Autoimmune thyroid disease; CD, Celiac disease; JIA, Juvenile idiopathic arthritis; RA, Rheumatoid arthritis; T1D, Type 1 diabetes.

### 5.2 Celiac disease

CD has obvious connections to gastrointestinal distress, gliadin peptides induce upregulation of zonulin and the shielding of gliadin peptides from destruction by lysosomes increases peptide secretion into the intestinal lamina propria, perpetuating inflammation and intestinal permeability (29, 63). Studies focusing on fecal microbiota show lower abundance of *Bifidobacterium* and *Lactobacillus* species in CD patients than healthy controls, both of which are considered to have anti-inflammatory effects (29, 104–106). Research from the Celiac Disease Genomic Environmental Microbiome and Metabolomic (CDGEMM) study, a prospective cohort of healthy infants with a first-degree relative who has CD, shows microbiome composition patterns up to 15 months before disease onset—increased abundance of *Dialister invisus, Parabacteroides* sp.*, Porphyromonas* sp., *Ruminococus bicirculans*, Lachnospiraceae and decreased abundance of *Streptococcus thermophilus, Faecalibacterium prausnitzii*, and *Clostridium clostridioforme* (107). Microbiome differences between those with a future diagnosis of CD and healthy matched controls can be seen as early as one year of age (41). Children progressed to CD diagnosis not only have a distinct microbiome composition compared to healthy controls but also have an increased IgA response, resulting in more IgA-coated bacteria, suggesting altered bacterial clearance (108). A recent study shows that changes in the gut microbiome, specifically abundance of Veillonellaceae, may have causal effects on CD development, while Pasteurellaceae abundance differences may be caused by the disease itself (109). Since CD occurs in the small intestine, intestinal location specific studies reveal the importance of location in microbial composition—higher abundance of *Escherichia coli*, *Prevotella salivae*, and *Neisseria* are associated with CD when sampling the duodenum (110).

### 5.3 Autoimmune thyroid disease

The gut-thyroid axis is a relatively new discussion point in study of autoimmune thyroid disease (AITD). Evidence that suggests dysbiosis is seen across AITD patients and the disruption of the gut microbial composition affects thyroid hormone metabolism (111, 112). Serum lipopolysaccharide and zonulin are significantly higher in GD patients than healthy controls and fecal transplant from GD patients into a mouse model significantly increases the incidence of GD (113, 114). However, specific microbiome community dynamics and potential for microbial biomarkers remains conflicted. The ratio of Firmicutes to Bacteroidetes is seen elevated in one study but significantly decreased in the AITD patients of a different study (115). Specifically, *Bacteroides fragilis* is observed as both higher and lower in abundance in AITD patients compared to healthy controls depending on the study (**Table 1**) (113, 115). *Lactobacillus* is proposed as both a potential probiotic for AITD amelioration and a potential major player in AITD pathogenesis (115, 116). Larger and more extensive microbiome studies may be required if a potential microbial biomarker for the gut-thyroid axis is determined.

### 5.4 Autoimmune arthritis

Despite emphasis on joint inflammation, the majority of RA patients also exhibit gastrointestinal disorders and significant gut microbiome differences are observed in RA patients versus controls (**Table 1**) (117–119). Patients with RA have a decreased microbial diversity and distinct microbial patterns (120). *Collinsella*, *Eggerthella*, and *Faecalibacterium* segregate with RA in random forest modeling (121) *Collinsella* and *Eggerthella* correlate with increased intestinal permeability, mucosal inflammation, and immune response, and *Collinsella* is confirmed to correlate with increased proinflammatory cytokine IL-17A, gut permeability, and RA disease severity (119, 121). Specifically, *Prevotella copri* is more abundant in new-onset RA patients, while *Bacteroides* and *Bifidobacterium* are decreased in the same population (120). *Prevotella* is also substantially more prevalent in children with a future diagnosis of JIA, while *Bifidobacterium* and *Bacteroides* species are reduced in the same cohort (86). A recent study in mice and colonic tissue shows increased intestinal permeability and zonulin-1 expression upon exposure to fecal bacteria from pre-RA human individuals (122). A recent small human RA study following bowel cleansing and fasting found a link between intestinal microbes and inflammation specific to RA, suggesting dysbiosis as a primary player in disease activity (123).

## 6 Evidence for HLA-associated dysbiosis

The fundamental role of class II HLA is to bind to foreign peptides and present them on the plasma membrane for recognition by CD4+ T helper cells. Within the gut, this antigen presentation leads to B cell production of secretory IgA. IgA mediates microbial composition by inhibiting bacterial adhesion to epithelial cells, regulating bacterial epitope expression, and facilitating the elimination of bacteria from the gut *via* peristaltic and mucociliary actions (124, 125). Structural variety associated with HLA allelic polymorphisms alters microbiome composition by linking MHC-peptide binding affinity differences to which bacteria get eliminated by IgA (36, 126, 127). With regard to gut microbiome composition, HLA polymorphisms significantly alter biases in antibody-mediated selection against microbiota and in turn correlate to unique microbial communities (14). Evidence shows that populations with functionally similar HLA also feature similar microbial patterns (128). Many studies group HLA haplotypes by autoimmune risk group. Therefore, most analysis available look at both DR3-DQ2 and DR4-DQ8 as either pooled homozygotes and/or heterozygotes. Future research could benefit from analyzing risk haplotypes against each other to verify their similarities and differences in influence over the microbiome. Compared to low-risk or neutral haplotypes, high-risk HLA DR3-DQ2 and DR4-DQ8 are associated with higher abundance of *Prevotella copri* at the species level, *Agathobacter, Bacteroides, Blautia, Dorea, Enterococcus, Intestinimonas, Klebsiella, Veillonella* at the genus level, and Enterobacteriaceae, which includes *Klebsiella*, Lachnospiraceae, which includes *Agathobacter*, *Blautia*, and *Dorea*, and Ruminococcaceae, which includes *Intestinimonas*, at the family level (36, 129–132). *Bifidobacterium* and *Lactobacillus* stand out as either negatively associated or in lower abundance in DR3-DQ2 and DR4-DQ8 compared to protective or neutral alleles (36, 39, 133, 134). Of note, the large general population cohort, All Babies in Southeat Sweden (ABIS), found that when controlling for breastfeeding, DR5-DQ7 is a significant factor in an infant’s likelihood to be colonized by *Lactobacillus* at all (135). This correlation may be associated with DQ7.5 *trans* configuration with DQ2.2 to create DQ2.5, the primary risk allele for CD. Aside from the association of increased relative abundance of *Bifidobacterium* in homozygous the DR1-DQ5 population (39), there has been limited examination into the role of DR1-DQ5 in microbiome community constructs to date.

## 7 Discussion

Many autoimmune disorders share the same risk-associated HLA haplotypes often resulting in comorbidity despite differing etiologies (22–24). The role of the gut microbiome has become increasingly essential in defining the pathogenesis of these autoimmune diseases (25–30). It has been theorized that dysbiosis seen in autoimmune diseases is associated with systemic inflammation, resulting in loss of barrier function and permeability of tight junctions, allowing for possible increased exposure of HLA proteins to bacterial antigens (31–33). HLA class II proteins are expressed in the upper villi of small intestinal enterocytes at a steady state in the presence of a healthy gut microbiome and are an integral part of maintaining homeostasis. However, dysbiosis and inflammation cause an increase in HLA class II expression in small intestinal crypts and the colonic epithelium, which can in turn influence the composition of the gut microbiome (32, 34–39). The fundamental role of class II HLA is to bind to foreign peptides and present them on the plasma membrane for recognition by CD4+ T helper cells. Within the gut, this antigen presentation leads to B cell production of secretory IgA. IgA mediates microbial composition by inhibiting bacterial adhesion to epithelial cells, regulating bacterial epitope expression, and facilitating the elimination of bacteria from the gut *via* peristaltic and mucociliary actions (124, 125). Structural differences associated with HLA allelic polymorphisms alter microbiome composition by linking HLA-peptide binding affinity differences to which bacteria get eliminated by IgA (36, 126, 127). The precedence for HLA molecular “preference” for specific peptides can be seen in celiac disease, where HLA DQ2 and DQ8 affinity for negatively charged residues results in class II MHC molecules binding and presenting gliadin peptides, leading to autoimmunity. However, certain HLA haplotypes, specifically the known risk HLA alleles discussed here, are associated with microbiome community dynamics that implicate dysbiosis before autoimmunity occurs (36, 39, 41, 42). Such evidence suggests that certain HLA may be predisposing an individual to systemic inflammation originating from the gut microbiome by clearing beneficial microbes and creating the potential for dysbiosis early in life. Increased exposure to commensal bacteria and excessive immune response over time could result in aberrant self-tolerance mechanisms.

Patterns emerge when investigating the overlap between HLA-associated and autoimmune-associated microbiomes. These relationships are unsurprising when considering the common risk-associated HLA haplotypes by autoimmunities. For example, *Prevotella copri* is more abundant in RA, JIA, and T1D patients compared to controls and is also associated HLA DR3-DQ2 and DR4-DQ8 (98, 99, 120, 131). This pattern makes sense when considering that HLA DR4-DQ8 is a risk-associated genotype for both RA and T1D. Higher abundances of inflammatory microbes, like *Klebsiella* and *Veillonella*, are associated with autoimmunity and risk; while conversely, lower abundances of known anti-inflammatory microbes like *Bifidobacterium* and *Lactobacillus* are associated with both autoimmune disorders and risk HLA (29, 104–106, 135–137). Causal relationships between microbiome composition and autoimmune onset are starting to be investigated. A recent study shows that abundance of Veillonellaceae may have causal effects on CD development (109). Prior to seroconversion, significantly higher abundance of *Bacteroides* species are observed in children with future T1D and JIA autoantibody seroconversion (37, 86). Both of these bacteria are associated with risk HLA (36, 138) Microbiome community differences can be seen as early as one year of age between those who go on to acquire an autoimmune disease versus those who do not (37, 41, 42). It is possible that the common denominator here is the introduction of early-life inflammation caused by HLA-specific dysbiosis.

It is important to note that all these studies focus on fecal microbiota, meaning the microbial composition is likely exclusively colonic and does not represent the small intestines. The field would benefit from microbial sampling from within a variety of locations in the gut. Also, there is limited current research into the gut-thyroid axis. Larger and more extensive microbiome studies may be required if a potential microbial biomarker for the gut-thyroid axis is to be determined. Many of the large cohort studies in this review focus on high-genetic-risk communities. To truly determine the impact of genetics on the gut, the field would benefit from general population studies that can compare risk vs. non-risk groups.

To validate the hypothesis that gut dysbiosis leads to early-life inflammation and elevate the link between gut microbiome composition and autoimmune disease onset, we propose an organ-on-a-chip model of human intestines. Within this model system, HLA-specific intestinal cultures could be generated to establish phenotypic differences between risk, neutral, and protection-associated tissue. To investigate innate immune response, we suggest quantifying cytokine secretion and examining zonulin, mucin, and permeability levels at baseline and following co-culture with either specific microbes of interest or a bacterial community culture (139, 140). It would be of interest to explore an adaptive immune response, as well. It is possible to characterize HLA-specific T cell response to commensal gut bacterial peptides through the presentation of secreted bacterial peptides to T cell stimulation assays measured with flow cytometry and ELISpot (141). An HLA-specific intestinal organ-on-a-chip model could also be used to measure T cell response by co-culturing peripheral blood mononuclear cells within the bottom chamber of the microfluidic chip and assessing T cell stimulation from the peptides that make it through the epithelial barrier in the microfluidic system.

The tripartite HLA-microbiome-autoimmunity link is not trivial. Risk HLA may be predisposing an individual early in life to dysbiosis originating in the clearance of beneficial microbes and/or promotion of inflammatory microbes, creating the potential systemic inflammation later in life. While it may be enticing to put emphasis on the dysbiosis and inflammation seen after autoimmune onset because of the clear evidence that autoimmune-induced permeability of tight junctions allows for increased exposure of HLA proteins to bacterial antigens, it is important to consider genetics and the initial role haplotype-specific peptide binding affinities may play in defining an individual’s microbiome.

## Author contributions

MB: Conceptualization, Data curation, Writing – original draft. ET: Conceptualization, Supervision, Writing – review & editing. JI: Supervision, Validation, Writing – review & editing. JL: Conceptualization, Project administration, Supervision, Writing – review & editing.

## References

[1] Trowsdale J Knight JC. Major histocompatibility complex genomics and human disease. Annu Rev Genomics Hum Genet (2013) 14:301–23. doi: 10.1146/annurev-genom-091212-153455 PMC4426292 23875801

[2] Dendrou CA Petersen J Rossjohn J Fugger L. HLA variation and disease. Nat Rev Immunol (2018) 18:325–39. doi: 10.1038/nri.2017.143 29292391

[3] Schott G Garcia-Blanco MA. MHC class III RNA binding proteins and immunity. RNA Biol (2021) 18(5):640–6. doi: 10.1080/15476286.2020.1860388 PMC8163431 33280511

[4] Miles JJ McCluskey J Rossjohn J Gras S. Understanding the complexity and malleability of T-cell recognition. Immunol Cell Biol (2015) 93:433–41. doi: 10.1038/icb.2014.112 25582337

[5] Singh NK Alonso JA Devlin JR Keller GLJ Gray GI Chiranjivi AK. A class-mismatched TCR bypasses MHC restriction *via* an unorthodox but fully functional binding geometry. Nat Commun (2022) 13:7189. doi: 10.1038/s41467-022-34896-0 36424374 PMC9691722

[6] Rist M Smith C Bell MJ Burrows SR Khanna R. Cross-recognition of HLA DR4 alloantigen by virus-specific CD8+ T cells: a new paradigm for self-/nonself-recognition. Blood (2009) 114:2244–53. doi: 10.1182/blood-2009-05-222596 19617574

[7] Abualrous ET Sticht J Freund C. Major histocompatibility complex (MHC) class I and class II proteins: impact of polymorphism on antigen presentation. Curr Opin Immunol (2021) 70:95–104. doi: 10.1016/j.coi.2021.04.009 34052735

[8] Djilali-Saiah I Benini V Daniel S Assan R Bach JF Caillat-Zucman S. Linkage disequilibrium between HLA class II (DR, DQ, DP) and antigen processing (LMP, TAP, DM) genes of the major histocompatibility complex. Tissue Antigens (1996) 48:87–92. doi: 10.1111/j.1399-0039.1996.tb02612.x 8883297

[9] Erlich H Valdes AM Noble J Carlson JA Varney M Concannon P. HLA DR-DQ haplotypes and genotypes and type 1 diabetes risk. Diabetes (2008) 57:1084–92. doi: 10.2337/db07-1331 PMC4103420 18252895

[10] Noble JA Erlich HA. Genetics of type 1 diabetes. Cold Spring Harb Perspect Med (2012) 2:a007732. doi: 10.1101/cshperspect.a007732 22315720 PMC3253030

[11] Noble JA Valdes AM. Genetics of the HLA region in the prediction of type 1 diabetes. Curr Diabetes Rep (2011) 11:533–42. doi: 10.1007/s11892-011-0223-x PMC3233362 21912932

[12] Jerram ST Leslie RD. The genetic architecture of type 1 diabetes. Genes (Basel) (2017) 8(8):209. doi: 10.3390/genes8080209 PMC5575672 28829396

[13] Rappazzo CG Huisman BD Birnbaum ME. Repertoire-scale determination of class II MHC peptide binding *via* yeast display improves antigen prediction. Nat Commun (2020) 11. doi: 10.1038/s41467-020-18204-2 PMC7473865 32887877

[14] Kubinak JL Stephens WZ Soto R Petersen C Chiaro T Gogokhia L. MHC variation sculpts individualized microbial communities that control susceptibility to enteric infection. Nat Commun (2015) 6. doi: 10.1038/ncomms9642 PMC4621775 26494419

[15] Atkinson MA Eisenbarth GS Michels AW. Type 1 diabetes. Lancet (2014) 383:69–82. doi: 10.1016/S0140-6736(13)60591-7 23890997 PMC4380133

[16] Bodis G Toth V Schwarting A. Role of human leukocyte antigens (HLA) in autoimmune diseases. Rheumatol Ther (2018) 5:5–20. doi: 10.1007/s40744-018-0100-z 29516402 PMC5935613

[17] Taka A-M But A Lempainen J Vatanen T Härkönen T Ilonen J. Finnish children carrying the high-risk HLA genotype have a 45-fold increased risk of type 1 diabetes compared to peers with neutral or protective genotypes. Diabetes Res Clin Pract (2023) 197:110256. doi: 10.1016/j.diabres.2023.110256 36640866

[18] Jacobson EM Huber A Tomer Y. THE HLA GENE COMPLEX IN THYROID AUTOIMMUNITY: FROM EPIDEMIOLOGY TO ETIOLOGY. J Autoimmun (2008) 30:58–62. doi: 10.1016/j.jaut.2007.11.010 18178059 PMC2244911

[19] Tuomilehto J. The emerging global epidemic of type 1 diabetes. Curr Diabetes Rep (2013) 13:795–804. doi: 10.1007/s11892-013-0433-5 24072479

[20] Lerner A Jeremias P Matthias T. The world incidence and prevalence of autoimmune diseases is increasing. Int J Celiac Dis (2015) 3:151–5. doi: 10.12691/ijcd-3-4-8

[21] Rewers M Ludvigsson J. Environmental risk factors for type 1 diabetes. Lancet (2016) 387:2340–8. doi: 10.1016/S0140-6736(16)30507-4 PMC5571740 27302273

[22] Alshiekh S Maziarz M Geraghty DE Larsson HE Agardh D. High-resolution genotyping indicates that children with type 1 diabetes and celiac disease share three HLA class II loci in DRB3, DRB4 and DRB5 genes. HLA (2021) 97:44–51. doi: 10.1111/tan.14105 33043613 PMC7756432

[23] Koning F Thomas R Rossjohn J Toes RE. Coeliac disease and rheumatoid arthritis: similar mechanisms, different antigens. Nat Rev Rheumatol (2015) 11:450–61. doi: 10.1038/nrrheum.2015.59 25986717

[24] Miyadera H Tokunaga K. Associations of human leukocyte antigens with autoimmune diseases: challenges in identifying the mechanism. J Hum Genet (2015) 60:697–702. doi: 10.1038/jhg.2015.100 26290149

[25] Heidt C Kämmerer U Fobker M Rüffer A Marquardt T Reuss-Borst M. Assessment of intestinal permeability and inflammation bio-markers in patients with rheumatoid arthritis. Nutrients (2023) 15:2386. doi: 10.3390/nu15102386 37242269 PMC10221762

[26] Piccioni A Rosa F Mannucci S Manca F Merra G Chiloiro S. Gut microbiota, LADA, and type 1 diabetes mellitus: an evolving relationship. Biomedicines (2023) 11:707. doi: 10.3390/biomedicines11030707 36979685 PMC10045633

[27] Monticolo M Mucha K Foroncewicz B. Lupus nephritis and dysbiosis. Biomedicines (2023) 11:1165. doi: 10.3390/biomedicines11041165 37189783 PMC10135948

[28] Knip M Siljander H. The role of the intestinal microbiota in type 1 diabetes mellitus. Nat Rev Endocrinol (2016) 12:154–67. doi: 10.1038/nrendo.2015.218 26729037

[29] Rossi RE Dispinzieri G Elvevi A Massironi S. Interaction between gut microbiota and celiac disease: from pathogenesis to treatment. Cells (2023) 12:823. doi: 10.3390/cells12060823 36980164 PMC10047417

[30] Xu Q Ni J-J Han B-X Yan S-S Wei X-T Feng G-J. Causal relationship between gut microbiota and autoimmune diseases: A two-sample mendelian randomization study. Front Immunol (2022) 12:746998. doi: 10.3389/fimmu.2021.746998 35140703 PMC8819003

[31] de Oliveira GLV Cardoso CRDB Taneja V Fasano A. Editorial: intestinal dysbiosis in inflammatory diseases. Front Immunol (2021) 12:727485. doi: 10.3389/fimmu.2021.727485 34394133 PMC8362080

[32] Davis-Richardson AG Triplett EW. A model for the role of gut bacteria in the development of autoimmunity for type 1 diabetes. Diabetologia (2015) 58:1386–93. doi: 10.1007/s00125-015-3614-8 PMC4473028 25957231

[33] Vaarala O. Leaking gut in type 1 diabetes. Curr Opin Gastroenterol (2008) 24:701–6. doi: 10.1097/MOG.0b013e32830e6d98 19122519

[34] Koyama M Mukhopadhyay P Schuster IS Henden AS Hülsdünker J Varelias A. MHC class II antigen presentation by the intestinal epithelium initiates graft-versus-host disease and is influenced by the microbiota. Immunity (2019) 51:885–898.e7. doi: 10.1016/j.immuni.2019.08.011 31542340 PMC6959419

[35] Wosen JE Mukhopadhyay D Macaubas C Mellins ED. Epithelial MHC class II expression and its role in antigen presentation in the gastrointestinal and respiratory tracts. Front Immunol (2018) 9:2144. doi: 10.3389/fimmu.2018.02144 30319613 PMC6167424

[36] Russell JT Roesch LFW Ördberg M Ilonen J Atkinson MA Schatz DA. Genetic risk for autoimmunity is associated with distinct changes in the human gut microbiome. Nat Commun (2019) 10:3621. doi: 10.1038/s41467-019-11460-x 31399563 PMC6689114

[37] Davis-Richardson AG Ardissone AN Dias R Simell V Leonard MT Kemppainen KM. Bacteroides dorei dominates gut microbiome prior to autoimmunity in Finnish children at high risk for type 1 diabetes. Front Microbiol (2014) 5:678. doi: 10.3389/fmicb.2014.00678 25540641 PMC4261809

[38] Murri M Leiva I Gomez-Zumaquero JM Tinahones FJ Cardona F Soriguer F. Gut microbiota in children with type 1 diabetes differs from that in healthy children: a case-control study. BMC Med (2013) 11:46. doi: 10.1186/1741-7015-11-46 23433344 PMC3621820

[39] Berryman MA Milletich PL Petrone JR Roesch LFW Ilonen J Triplett EW. Autoimmune-associated genetics impact probiotic colonization of the infant gut. J Autoimmun (2022) 133:102943. doi: 10.1016/j.jaut.2022.102943 36356550

[40] Arnaud-Battandier F Cerf-Bensussan N Amsellem R Schmitz J. Increased HLA-DR expression by enterocytes in children with celiac disease. Gastroenterology (1986) 91:1206–12. doi: 10.1016/s0016-5085(86)80018-x 3758613

[41] Milletich PL Ahrens AP Petrone JR Russell JT Berryman MA Agardh D. Gut microbiome markers in subgroups of HLA class II genotyped infants signal future celiac disease in the general population: ABIS study. Front Microbiol (2022) 12. doi: 10.3389/fcimb.2022.920735 PMC9357981 35959362

[42] Bélteky M Milletich PL Ahrens AP Triplett EW Ludvigsson J. Infant gut microbiome composition correlated with type 1 diabetes acquisition in the general population: the ABIS study. Diabetologia (2023) 66:1116–28. doi: 10.1007/s00125-023-05895-7 36964264

[43] Bondinas GP Moustakas AK Papadopoulos GK. The spectrum of HLA-DQ and HLA-DR alleles, 2006: a listing correlating sequence and structure with function. Immunogenetics (2007) 59:539–53. doi: 10.1007/s00251-007-0224-8 17497145

[44] Pinet V Eliaou JF Clot J. Description of a polymorphism in the regulatory region of the HLA-DRA gene. Hum Immunol (1991) 32:162–9. doi: 10.1016/0198-8859(91)90052-b 1685491

[45] Nilsson JB Kaabinejadian S Yari H Peters B Barra C Gragert L. Machine learning reveals limited contribution of trans-only encoded variants to the HLA-DQ immunopeptidome. Commun Biol (2023) 6:442. doi: 10.1038/s42003-023-04749-7 37085710 PMC10121683

[46] Tollefsen S Hotta K Chen X Simonsen B Swaminathan K Mathews II. Structural and functional studies of trans-encoded HLA-DQ2.3 (DQA1*03:01/DQB1*02:01) protein molecule. J Biol Chem (2012) 287:13611–9. doi: 10.1074/jbc.M111.320374 PMC3340161 22362761

[47] Gregory GA Robinson TIG Linklater SE Wang F Colagiuri S de Beaufort C. Global incidence, prevalence, and mortality of type 1 diabetes in 2021 with projection to 2040: a modelling study. Lancet Diabetes Endocrinol (2022) 10:741–60. doi: 10.1016/S2213-8587(22)00218-2 36113507

[48] Robertson CC Rich SS. Genetics of type 1 diabetes. Curr Opin Genet Dev (2018) 50:7–16. doi: 10.1016/j.gde.2018.01.006 29453110

[49] Ilonen J Kiviniemi M Lempainen J Simell O Toppari J Veijola R. Genetic susceptibility to type 1 diabetes in childhood – estimation of HLA class II associated disease risk and class II effect in various phases of islet autoimmunity. Pediatr Diabetes (2016) 17:8–16. doi: 10.1111/pedi.12327 27411431

[50] Kawabata Y Ikegami H Kawaguchi Y Fujisawa T Shintani M Ono M. Asian-specific HLA haplotypes reveal heterogeneity of the contribution of HLA-DR and -DQ haplotypes to susceptibility to type 1 diabetes. Diabetes (2002) 51:545–51. doi: 10.2337/diabetes.51.2.545 11812768

[51] Hu CY Allen M Chuang LM Lin BJ Gyllensten U. Association of insulin-dependent diabetes mellitus in Taiwan with HLA class II DQB1 and DRB1 alleles. Hum Immunol (1993) 38:105–14. doi: 10.1016/0198-8859(93)90526-7 8106265

[52] Krischer JP Lynch KF Lernmark Å Hagopian WA Rewers MJ She J-X. Genetic and environmental interactions modify the risk of diabetes-related autoimmunity by 6 years of age: the TEDDY study. Diabetes Care (2017) 40:1194–202. doi: 10.2337/dc17-0238 PMC5566280 28646072

[53] Fava D Gardner S Pyke D Leslie RD. Evidence that the age at diagnosis of IDDM is genetically determined. Diabetes Care (1998) 21:925–9. doi: 10.2337/diacare.21.6.925 9614609

[54] Mikk M-L Pfeiffer S Kiviniemi M Laine A-P Lempainen J Härkönen T. HLA-DR-DQ haplotypes and specificity of the initial autoantibody in islet specific autoimmunity. Pediatr Diabetes (2020) 21:1218–26. doi: 10.1111/pedi.13073 32613719

[55] Regnell SE Lernmark Å. Early prediction of autoimmune (type 1) diabetes. Diabetologia (2017) 60:1370–81. doi: 10.1007/s00125-017-4308-1 PMC5491594 28550517

[56] Zhao LP Papadopoulos GK Lybrand TP Moustakas AK Bondinas GP Carlsson A. The KAG motif of HLA-DRB1 (β71, β74, β86) predicts seroconversion and development of type 1 diabetes. EBioMedicine (2021) 69:103431. doi: 10.1016/j.ebiom.2021.103431 34153873 PMC8220560

[57] Zhao LP Papadopoulos GK Kwok WW Moustakas AK Bondinas GP Larsson HE. Motifs of three HLA-DQ amino acid residues (α44, β57, β135) capture full association with the risk of type 1 diabetes in DQ2 and DQ8 children. Diabetes (2020) 69:1573–87. doi: 10.2337/db20-0075 PMC7306123 32245799

[58] Hu X Deutsch AJ Lenz TL Onengut-Gumuscu S Han B Chen W-M. Additive and interaction effects at three amino acid positions in HLA-DQ and HLA-DR molecules drive type 1 diabetes risk. Nat Genet (2015) 47:898–905. doi: 10.1038/ng.3353 26168013 PMC4930791

[59] Chuzho N Mishra N Tandon N Kanga U Mishra G Sharma A. HLA-DR3 mediated CD4 T cell response against GAD65 in type 1 diabetes patients. J Diabetes (2023) 15:607–21. doi: 10.1111/1753-0407.13406 PMC10345980 37309552

[60] Rønningen K S Iwe T Halstensen TS Spurkland A Thorsby E. The amino acid at position 57 of the HLA-DQB chain and susceptibility to develop insulin-dependent diabetes mellitus. Hum Immunol (1989) 26:215–25. doi: 10.1016/0198-8859(89)90040-2 2606746

[61] Sollid LM. The roles of MHC class II genes and post-translational modification in celiac disease. Immunogenetics (2017) 69:605–16. doi: 10.1007/s00251-017-0985-7 28695286

[62] Lundin KE Scott H Fausa O Thorsby E Sollid LM. T cells from the small intestinal mucosa of a DR4, DQ7/DR4, DQ8 celiac disease patient preferentially recognize gliadin when presented by DQ8. Hum Immunol (1994) 41:285–91. doi: 10.1016/0198-8859(94)90047-7 7883596

[63] Qiao S-W Sollid LM Blumberg RS. Antigen presentation in celiac disease. Curr Opin Immunol (2009) 21:111. doi: 10.1016/j.coi.2009.03.004 19342211 PMC3901576

[64] Silvester JA Therrien A Kelly CP. Celiac disease: fallacies and facts. Am J Gastroenterol (2021) 116:1148–55. doi: 10.14309/ajg.0000000000001218 PMC8462980 33767109

[65] Krigel A Turner KO Makharia GK Green PH Genta RM Lebwohl B. Ethnic variations in duodenal villous atrophy consistent with celiac disease in the United States. Clin Gastroenterol Hepatol (2016) 14:1105–11. doi: 10.1016/j.cgh.2016.04.032 PMC4955830 27155557

[66] Banerjee P Chaudhary R Singh AK Parulekar P Kumar S Senapati S. Specific genetic polymorphisms contributing in differential binding of gliadin peptides to HLA-DQ and TCR to elicit immunogenicity in celiac disease. Biochem Genet (2023). doi: 10.1007/s10528-023-10377-x 37103600

[67] Ramakrishna BS Venugopal G Singh A Pugazhendhi S Dutta S Ahuja V. Human Leukocyte Antigen DQ (HLA-DQ) genotypes and haplotypes and their association with phenotype in patients with celiac disease in India. JGH Open (2021) 5:1190–6. doi: 10.1002/jgh3.12651 PMC8485407 34622007

[68] Piancatelli D Ben El Barhdadi I Oumhani K Sebastiani P Colanardi A Essaid A. HLA typing and celiac disease in Moroccans. Med Sci (Basel) (2017) 5:2. doi: 10.3390/medsci5010002 29099018 PMC5635774

[69] Almeida A Mitchell AL Boland M Forster SC Gloor GB Tarkowska A. A new genomic blueprint of the human gut microbiota. Nature (2019) 568:499–504. doi: 10.1038/s41586-019-0965-1 30745586 PMC6784870

[70] Pisapia L Picascia S Farina F Barba P Gianfrani C Del Pozzo G. Differential expression of predisposing HLA-DQ2.5 alleles in DR5/DR7 celiac disease patients affects the pathological immune response to gluten. Sci Rep (2020) 10:17227. doi: 10.1038/s41598-020-73907-2 33057065 PMC7560598

[71] Zeitlin AA Heward JM Newby PR Carr-Smith JD Franklyn JA Gough SCL. Analysis of HLA class II genes in Hashimoto’s thyroiditis reveals differences compared to Graves’ disease. Genes Immun (2008) 9:358–63. doi: 10.1038/gene.2008.26 18449200

[72] Simmonds MJ Howson JMM Heward JM Cordell HJ Foxall H Carr-Smith J. Regression mapping of association between the human leukocyte antigen region and graves disease. Am J Hum Genet (2005) 76:157–63. doi: 10.1086/426947 PMC1196419 15558498

[73] Heward JM Allahabadia A Daykin J Carr-Smith J Daly A Armitage M. Linkage disequilibrium between the human leukocyte antigen class II region of the major histocompatibility complex and graves’ Disease: replication using a population case control and family-based study1. J Clin Endocrinol Metab (1998) 83:3394–7. doi: 10.1210/jcem.83.10.5137 9768636

[74] Li CW Osman R Menconi F Hou H Schechter C Kozhakhmetova A. Effective inhibition of thyroid antigen presentation using retro-inverso peptides in experimental autoimmune thyroiditis: A pathway toward immune therapies of thyroid autoimmunity. Thyroid (2023) 33:492–500. doi: 10.1089/thy.2022.0511 36762945 PMC10325802

[75] Lee HJ Stefan-Lifshitz M Li CW Tomer Y. GENETICS AND EPIGENETICS OF AUTOIMMUNE THYROID DISEASES: TRANSLATIONAL IMPLICATIONS. Best Pract Res Clin Endocrinol Metab (2023) 37:101661. doi: 10.1016/j.beem.2022.101661 35459628 PMC9550878

[76] Nepom GT Byers P Seyfried C Healey LA Wilske KR Stage D. HLA genes associated with rheumatoid arthritis. Identification of susceptibility alleles using specific oligonucleotide probes. Arthritis Rheum (1989) 32:15–21. doi: 10.1002/anr.1780320104 2492197

[77] Gregersen PK Silver J Winchester RJ. The shared epitope hypothesis. an approach to understanding the molecular genetics of susceptibility to rheumatoid arthritis. Arthritis Rheumatism (1987) 30:1205–13. doi: 10.1002/art.1780301102 2446635

[78] Wang M Wu J Lei S Mo X. Genome-wide identification of RNA modification-related single nucleotide polymorphisms associated with rheumatoid arthritis. BMC Genomics (2023) 24:153. doi: 10.1186/s12864-023-09227-2 36973646 PMC10045113

[79] de Moel EC Derksen VFAM Trouw LA Bang H Collée G Lard LR. In rheumatoid arthritis, changes in autoantibody levels reflect intensity of immunosuppression, not subsequent treatment response. Arthritis Res Ther (2019) 21:28. doi: 10.1186/s13075-019-1815-0 30658699 PMC6339446

[80] Dessen A Lawrence CM Cupo S Zaller DM Wiley DC. X-ray crystal structure of HLA-DR4 (DRA*0101, DRB1*0401) complexed with a peptide from human collagen II. Immunity (1997) 7:473–81. doi: 10.1016/S1074-7613(00)80369-6 9354468

[81] Roudier J. HLA-DRB1 genes and extraarticular rheumatoid arthritis. Arthritis Res Ther (2006) 8:103. doi: 10.1186/ar1886 16542468 PMC1526581

[82] Jawaheer D Thomson W Macgregor AJ Carthy D Davidson J Dyer PA. “Homozygosity” for the HLA–DR shared epitope contributes the highest risk for rheumatoid arthritis concordance in identical twins. Arthritis Rheumatism (1994) 37:681–6. doi: 10.1002/art.1780370511 7514412

[83] Salesi M Boroujeni GT Salehi M Farzamnia S. Evaluation of differences in HLA-DR4 gene and its subtypes prevalence among healthy people and RA patients in Isfahan province population. Adv BioMed Res (2016) 5:11. doi: 10.4103/2277-9175.175244 26962513 PMC4770612

[84] Tesolin P Bertinetto FE Sonaglia A Cappellani S Concas MP Morgan A. High throughput genetic characterisation of caucasian patients affected by multi-drug resistant rheumatoid or psoriatic arthritis. J Pers Med (2022) 12:1618. doi: 10.3390/jpm12101618 36294757 PMC9605087

[85] Raychaudhuri S Sandor C Stahl EA Freudenberg J Lee H-S Jia X. Five amino acids in three HLA proteins explain most of the association between MHC and seropositive rheumatoid arthritis. Nat Genet (2012) 44:291–6. doi: 10.1038/ng.1076 PMC3288335 22286218

[86] Kindgren E Ahrens AP Triplett EW Ludvigsson J. Infant gut microbiota and environment associate with juvenile idiopathic arthritis many years prior to disease onset, especially in genetically vulnerable children. eBioMedicine (2023) 93:104654. doi: 10.1016/j.ebiom.2023.104654 PMC10279551 37329576

[87] Liao KP Gunnarsson M Källberg H Ding B Plenge RM Padyukov L. A specific association exists between type 1 diabetes and anti-CCP positive rheumatoid arthritis. Arthritis rheumatism (2009) 60:653. doi: 10.1002/art.24362 19248096 PMC2768389

[88] Triolo TM Armstrong TK McFann K Yu L Rewers MJ Klingensmith GJ. Additional autoimmune disease found in 33% of patients at type 1 diabetes onset. Diabetes Care (2011) 34:1211–3. doi: 10.2337/dc10-1756 PMC3114477 21430083

[89] Department of Pediatric Gastroenterology Adana City Training and Research Hospital, Adana, Turkey Gulcu Taskin D Ata A Department of Pediatric Endocrinology, Adana City Training and Research Hospital, Adana, Turkey. The screening of celiac serology in pediatric patients diagnosed with type 1 diabetes mellitus. Turk J Gastroenterol (2023) 34:293–7. doi: 10.5152/tjg.2023.22775 PMC10152150 36919834

[90] Kurppa K Laitinen A Agardh D. Coeliac disease in children with type 1 diabetes. Lancet Child Adolesc Health (2018) 2:133–43. doi: 10.1016/S2352-4642(17)30172-4 30169235

[91] Roy A Laszkowska M Sundström J Lebwohl B Green PHR Kämpe O. Prevalence of celiac disease in patients with autoimmune thyroid disease: A meta-analysis. Thyroid® (2016) 26:880–90. doi: 10.1089/thy.2016.0108 27256300

[92] Franco J-S Amaya-Amaya J Anaya J-M. Thyroid disease and autoimmune diseases, in: Autoimmunity: from bench to bedside (2013). El Rosario University Press. Available at: https://www.ncbi.nlm.nih.gov/books/NBK459466/ (Accessed July 19, 2023). 29087650

[93] Dore MP Fanciulli G Rouatbi M Mereu S Pes GM. Autoimmune thyroid disorders are more prevalent in patients with celiac disease: A retrospective case-control study. J Clin Med (2022) 11:6027. doi: 10.3390/jcm11206027 36294348 PMC9605329

[94] Doyle JB Lebwohl B Askling J Forss A Green PHR Roelstraete B. Risk of juvenile idiopathic arthritis and rheumatoid arthritis in patients with celiac disease: A population-based cohort study. Off J Am Coll Gastroenterol | ACG (2022) 117:1971. doi: 10.14309/ajg.0000000000002014 36114769

[95] Higuchi BS Rodrigues N Gonzaga MI Paiolo JCC Stefanutto N Omori WP. Intestinal dysbiosis in autoimmune diabetes is correlated with poor glycemic control and increased interleukin-6: A pilot study. Front Immunol (2018) 9:1689. doi: 10.3389/fimmu.2018.01689 30090100 PMC6068285

[96] Kemppainen KM Ardissone AN Davis-Richardson AG Fagen JR Gano KA León-Novelo LG. Early childhood gut microbiomes show strong geographic differences among subjects at high risk for type 1 diabetes. Diabetes Care (2015) 38:329–32. doi: 10.2337/dc14-0850 PMC4302256 25519450

[97] Giongo A Gano KA Crabb DB Mukherjee N Novelo LL Casella G. Toward defining the autoimmune microbiome for type 1 diabetes. ISME J (2011) 5:82–91. doi: 10.1038/ismej.2010.92 20613793 PMC3105672

[98] Stewart CJ Ajami NJ O’Brien JL Hutchinson DS Smith DP Wong MC. Temporal development of the gut microbiome in early childhood from the TEDDY study. Nature (2018) 562:583–8. doi: 10.1038/s41586-018-0617-x PMC6415775 30356187

[99] Traversi D Scaioli G Rabbone I Carletto G Ferro A Franchitti E. Gut microbiota, behavior, and nutrition after type 1 diabetes diagnosis: A longitudinal study for supporting data in the metabolic control. Front Nutr (2022) 9:968068. doi: 10.3389/fnut.2022.968068 36562032 PMC9763620

[100] Matos J Matos I Calha M Santos P Duarte I Cardoso Y. Insights from bacteroides species in children with type 1 diabetes. Microorganisms (2021) 9:1436. doi: 10.3390/microorganisms9071436 34361871 PMC8306409

[101] Bosi E Molteni L Radaelli MG Folini L Fermo I Bazzigaluppi E. Increased intestinal permeability precedes clinical onset of type 1 diabetes. Diabetologia (2006) 49:2824–7. doi: 10.1007/s00125-006-0465-3 17028899

[102] Vaarala O Atkinson MA Neu J. The “Perfect storm” for type 1 diabetes. Diabetes (2008) 57:2555–62. doi: 10.2337/db08-0331 PMC2551660 18820210

[103] Lo Conte M Cosorich I Ferrarese R Antonini Cencicchio M Nobili A Palmieri V. Alterations of the intestinal mucus layer correlate with dysbiosis and immune dysregulation in human Type 1 Diabetes. EBioMedicine (2023) 91:104567. doi: 10.1016/j.ebiom.2023.104567 37062177 PMC10139895

[104] Sanz Y Sánchez E Marzotto M Calabuig M Torriani S Dellaglio F. Differences in faecal bacterial communities in coeliac and healthy children as detected by PCR and denaturing gradient gel electrophoresis. FEMS Immunol Med Microbiol (2007) 51:562–8. doi: 10.1111/j.1574-695X.2007.00337.x 17919298

[105] Lorenzo Pisarello MJ Vintiñi EO González SN Pagani F Medina MS. Decrease in lactobacilli in the intestinal microbiota of celiac children with a gluten-free diet, and selection of potentially probiotic strains. Can J Microbiol (2015) 61:32–7. doi: 10.1139/cjm-2014-0472 25438612

[106] Olivares M Benítez-Páez A de Palma G Capilla A Nova E Castillejo G. Increased prevalence of pathogenic bacteria in the gut microbiota of infants at risk of developing celiac disease: The PROFICEL study. Gut Microbes (2018) 9:551–8. doi: 10.1080/19490976.2018.1451276 PMC6287676 29672211

[107] Leonard MM Valitutti F Karathia H Pujolassos M Kenyon V Fanelli B. Microbiome signatures of progression toward celiac disease onset in at-risk children in a longitudinal prospective cohort study. Proc Natl Acad Sci U.S.A. (2021) 118:e2020322118. doi: 10.1073/pnas.2020322118 34253606 PMC8307711

[108] Girdhar K Dogru YD Huang Q Yang Y Tolstikov V Raisingani A. Dynamics of the gut microbiome, IgA response, and plasma metabolome in the development of pediatric celiac disease. Microbiome (2023) 11:9. doi: 10.1186/s40168-022-01429-2 36639805 PMC9840338

[109] González-García BP Marí S Cilleros-Portet A Hernangomez-Laderas A Fernandez-Jimenez N García-Santisteban I. Two-Sample Mendelian Randomization detects bidirectional causality between gut microbiota and celiac disease in individuals with high genetic risk. Front Immunol (2023) 14:1082862. doi: 10.3389/fimmu.2023.1082862 37457693 PMC10347381

[110] Constante M Libertucci J Galipeau HJ Szamosi JC Rueda G Miranda PM. Biogeographic variation and functional pathways of the gut microbiota in celiac disease. Gastroenterology (2022) 163:1351–1363.e15. doi: 10.1053/j.gastro.2022.06.088 35810781

[111] Knezevic J Starchl C Tmava Berisha A Amrein K. Thyroid-gut-axis: how does the microbiota influence thyroid function? Nutrients (2020) 12:1769. doi: 10.3390/nu12061769 32545596 PMC7353203

[112] Fenneman AC Bruinstroop E Nieuwdorp M van der Spek AH Boelen A. A comprehensive review of thyroid hormone metabolism in the gut and its clinical implications. Thyroid® (2023) 33:32–44. doi: 10.1089/thy.2022.0491 36322786

[113] Su X Yin X Liu Y Yan X Zhang S Wang X. Gut dysbiosis contributes to the imbalance of treg and th17 cells in graves’ Disease patients by propionic acid. J Clin Endocrinol Metab (2020) 105:3526–47. doi: 10.1210/clinem/dgaa511 32785703

[114] Zheng D Liao H Chen S Liu X Mao C Zhang C. Elevated levels of circulating biomarkers related to leaky gut syndrome and bacterial translocation are associated with graves’ Disease. Front Endocrinol (Lausanne) (2021) 12:796212. doi: 10.3389/fendo.2021.796212 34975767 PMC8716831

[115] Gong B Wang C Meng F Wang H Song B Yang Y. Association between gut microbiota and autoimmune thyroid disease: A systematic review and meta-analysis. Front Endocrinol (Lausanne) (2021) 12:774362. doi: 10.3389/fendo.2021.774362 34867823 PMC8635774

[116] Jiang W Yu X Kosik RO Song Y Qiao T Tong J. Gut microbiota may play a significant role in the pathogenesis of graves’ Disease. Thyroid (2021) 31:810–20. doi: 10.1089/thy.2020.0193 PMC8110022 33234057

[117] Terato K Do CT Shionoya H. Slipping through the cracks: linking low immune function and intestinal bacterial imbalance to the etiology of rheumatoid arthritis. Autoimmune Dis (2015) 2015:e636207. doi: 10.1155/2015/636207 PMC4377354 25861466

[118] Chen J Li S Zhu J Su W Jian C Zhang J. Multi-omics profiling reveals potential alterations in rheumatoid arthritis with different disease activity levels. Arthritis Res Ther (2023) 25:74. doi: 10.1186/s13075-023-03049-z 37138305 PMC10155393

[119] Blenkinsopp HC Seidler K Barrow M. Microbial imbalance and intestinal permeability in the pathogenesis of rheumatoid arthritis: A mechanism review with a focus on bacterial translocation, citrullination, and probiotic intervention. J Am Nutr Assoc (2023), 1–18. doi: 10.1080/27697061.2023.2211129 37294082

[120] Meng X Zhou H-Y Shen H-H Lufumpa E Li X-M Guo B. Microbe-metabolite-host axis, two-way action in the pathogenesis and treatment of human autoimmunity. Autoimmun Rev (2019) 18:455–75. doi: 10.1016/j.autrev.2019.03.006 30844549

[121] Chen J Wright K Davis JM Jeraldo P Marietta EV Murray J. An expansion of rare lineage intestinal microbes characterizes rheumatoid arthritis. Genome Med (2016) 8:43. doi: 10.1186/s13073-016-0299-7 27102666 PMC4840970

[122] Luo Y Tong Y Wu L Niu H Li Y Su LC. Alteration of gut microbiota in high-risk individuals for rheumatoid arthritis is associated with disturbed metabolome and initiates arthritis by triggering mucosal immunity imbalance. Arthritis Rheumatol (2023). doi: 10.1002/art.42616 37219936

[123] Häupl T Sörensen T Smiljanovic B Darcy M Scheder-Bieschin J Steckhan N. Intestinal microbiota reduction followed by fasting discloses microbial triggering of inflammation in rheumatoid arthritis. J Clin Med (2023) 12:4359. doi: 10.3390/jcm12134359 37445394 PMC10342944

[124] Mantis NJ Rol N Corthésy B. Secretory IgA’s complex roles in immunity and mucosal homeostasis in the gut. Mucosal Immunol (2011) 4:603–11. doi: 10.1038/mi.2011.41 PMC3774538 21975936

[125] Peterson LW Artis D. Intestinal epithelial cells: regulators of barrier function and immune homeostasis. Nat Rev Immunol (2014) 14:141–53. doi: 10.1038/nri3608 24566914

[126] Roland MM Mohammed AD Kubinak JL. How MHCII signaling promotes benign host-microbiota interactions. PloS Pathog (2020) 16. doi: 10.1371/journal.ppat.1008558 PMC7323949 32598378

[127] Silverman M Kua L Tanca A Pala M Palomba A Tanes C. Protective major histocompatibility complex allele prevents type 1 diabetes by shaping the intestinal microbiota early in ontogeny. Proc Natl Acad Sci U.S.A. (2017) 114:9671–6. doi: 10.1073/pnas.1712280114 PMC5594701 28831005

[128] Andeweg SP Keşmir C Dutilh BE. Quantifying the impact of human leukocyte antigen on the human gut microbiota. mSphere (2021) 6:e00476–21. doi: 10.1128/mSphere.00476-21 PMC8386457 34378979

[129] Leonard MM Karathia H Pujolassos M Troisi J Valitutti F Subramanian P. Multi-omics analysis reveals the influence of genetic and environmental risk factors on developing gut microbiota in infants at risk of celiac disease. Microbiome (2020) 8:130. doi: 10.1186/s40168-020-00906-w 32917289 PMC7488762

[130] Aguayo-Patrón SV Trujillo-Rivera OA Cornejo-Granados F Ochoa-Leyva A Calderón de la Barca AM. HLA-haplotypes influence microbiota structure in northwestern mexican schoolchildren predisposed for celiac disease or type 1 diabetes. Microorganisms (2023) 11:1412. doi: 10.3390/microorganisms11061412 37374914 PMC10305193

[131] Yue T Tan H Wang C Liu Z Yang D Ding Y. High-risk genotypes for type 1 diabetes are associated with the imbalance of gut microbiome and serum metabolites. Front Immunol (2022) 13. doi: 10.3389/fimmu.2022.1033393 PMC9794034 36582242

[132] Clancy RM Marion MC Ainsworth HC Chang M Howard TD Izmirly PM. Gut dysbiosis and the clinical spectrum in anti-Ro positive mothers of children with neonatal lupus. Gut Microbes (2022) 14:2081474. doi: 10.1080/19490976.2022.2081474 35704681 PMC9225419

[133] Olivares M Neef A Castillejo G Palma GD Varea V Capilla A. The HLA-DQ2 genotype selects for early intestinal microbiota composition in infants at high risk of developing coeliac disease. Gut (2015) 64:406–17. doi: 10.1136/gutjnl-2014-306931 24939571

[134] Vatanen T Franzosa EA Schwager R Tripathi S Arthur TD Vehik K. The human gut microbiome in early-onset type 1 diabetes from the TEDDY study. Nature (2018) 562:589–94. doi: 10.1038/s41586-018-0620-2 PMC6296767 30356183

[135] Berryman MA Triplett EW Ludvigsson J. Human leucocyte antigen-dependent colonization of lactobacillus in the early-life gut. Front Microbiomes (2023) 2:1192773. doi: 10.3389/frmbi.2023.1192773 PMC12993612 41853370

[136] Rashid T Ebringer A Wilson C. The role of *Klebsiella* in Crohn’s disease with a potential for the use of antimicrobial measures. Int J Rheumatol (2013) 2013:e610393. doi: 10.1155/2013/610393 PMC3810322 24223596

[137] Zhan Z Liu W Pan L Bao Y Yan Z Hong L. Overabundance of Veillonella parvula promotes intestinal inflammation by activating macrophages *via* LPS-TLR4 pathway. Cell Death Discovery (2022) 8:251. doi: 10.1038/s41420-022-01015-3 35523778 PMC9076897

[138] Endesfelder D Engel M Davis-Richardson AG Ardissone AN Achenbach P Hummel S. Towards a functional hypothesis relating anti-islet cell autoimmunity to the dietary impact on microbial communities and butyrate production. Microbiome (2016) 4:17. doi: 10.1186/s40168-016-0163-4 27114075 PMC4845316

[139] Šuligoj T Vigsnæs LK den Abbeele PV Apostolou A Karalis K Savva GM. Effects of human milk oligosaccharides on the adult gut microbiota and barrier function. Nutrients (2020) 12:2808. doi: 10.3390/nu12092808 32933181 PMC7551690

[140] Pediaditakis I Kodella KR Manatakis DV Le CY Barthakur S Sorets A. A microengineered Brain-Chip to model neuroinflammation in humans. iScience (2022) 25. doi: 10.1016/j.isci.2022.104813 PMC9379671 35982785

[141] Ogbe A Kronsteiner B Skelly DT Pace M Brown A Adland E. T cell assays differentiate clinical and subclinical SARS-CoV-2 infections from cross-reactive antiviral responses. Nat Commun (2021) 12. doi: 10.1038/s41467-021-21856-3 PMC8024333 33824342

